# Genome Sequence of a Small RNA Virus of the Southern Corn Rootworm, Diabrotica undecimpunctata howardi Barber (Coleoptera: Chrysomelidae)

**DOI:** 10.1128/MRA.00379-20

**Published:** 2020-06-25

**Authors:** Sijun Liu, Arnubio Valencia-Jiménez, Molly Darlington, Ana M. Vélez, Bryony C. Bonning

**Affiliations:** a Department of Entomology, Iowa State University, Ames, Iowa, USA; b Departamento de Producción Agropecuaria, Universidad de Caldas, Manizales, Colombia; c Department of Entomology, University of Nebraska—Lincoln, Lincoln, Nebraska, USA; d Department of Entomology and Nematology, University of Florida, Gainesville, Florida, USA; Portland State University

## Abstract

The genome sequence of a novel small RNA virus, tentatively named *Diabrotica undecimpunctata virus 1* (DuV1), was discovered from the transcriptome of the southern corn rootworm, Diabrotica undecimpunctata *howardi* Barber. DuV1 has a positive-sense, single-stranded RNA genome that encodes a single polyprotein of 3,401 amino acids with limited similarity to other viruses.

## ANNOUNCEMENT

The southern corn rootworm (SCR), Diabrotica undecimpunctata howardi Barber, is a polyphagous pest that impacts multiple crops in the southeastern United States, southern Canada, and northern Mexico (1). While several small RNA viruses have been reported from other rootworm species (2–4), this is the first report of a virus genome sequence from the transcriptome of *D. undecimpunctata*.

SCR were supplied by Crop Characteristics (Farmington, MN). Total RNA was isolated from three biological replicates of multiple developmental stages (eggs; first-, second-, and third-instar larvae; pupae; adult females; and adult males) using the RNeasy minikit (Qiagen, Valencia, CA, USA). RNA extractions from each stage-specific sample were used for cDNA library construction with poly(A) selection. The NEBNext Ultra RNA library prep kit for Illumina was used for library preparation. cDNA libraries were paired-end sequenced (150-nucleotide [nt] reads) on a HiSeq 2500 platform (Illumina, San Diego, CA). High-quality sequencing reads (48.2 million reads for males, 41.5 million reads for females; mean quality score, 37.5) were assembled by using the Trinity assembler v2.6.6 (5). Default parameters were used for all software unless otherwise noted. Putative viral sequences were initially identified by BLASTx analysis (6) against a local viral sequence database with the following cutoff values: contig length, >300 nt; protein sequence length, >50 amino acids [aa]; and E value, 0.01. Here, we describe one of two near-complete viral genome sequences identified from this analysis.

A 10,483-nt contig with a G+C content of 47% was assembled from the sequencing reads from both female and male adult SCR and was named *Diabrotica undecimpunctata virus 1* (DuV1). A total of 1,070 reads from males and 1,078 from females mapped to viral sequences with a coverage of 15.3 and 15.4, respectively. Only a few short fragments were identified from larval samples. A 10,203-nt coding region was flanked by a 5′ untranslated region (UTR) of at least 139 nt and a 3′ UTR of at least 141 nt. The predicted molecular mass of the polyprotein is 378.7 kDa. The N-terminal half of the hypothetical protein contains putative nonstructural proteins, with the C-terminal half comprised of putative capsid proteins. The nonstructural protein sequence contained six conserved domains, four of which overlapped, i.e., Macro_Poa1p_like domain (aa 46 to 151; NCBI accession no. cd02901); A1pp, an appr-1″-p processing enzyme (aa 48 to 149; SMART accession no. SM00506); Macro, an ADP-ribose binding module (aa 89 to 149; NCBI accession no. pfam01661); and Ymd, an *O*-acetyl-ADP-ribose deacetylase (regulator of RNase III) (aa 63 to 174; NCBI accession no. COG2110). An RNA_helicase domain was located between aa 812 and 913 (NCBI accession no. pfam00910), and an RdRP domain was located between aa 1934 and 2367 (EMBL-EBI accession no. pfam00680, NCBI accession no. cd01699). Two cysteine protease sequence motifs, GxCG and GxHxxG, were identified between aa 1734 and 1737 (GDCG) and aa 1755 and 1760 (GIHVAG). No conserved domains were found in the putative viral capsid region.

**FIG 1:**
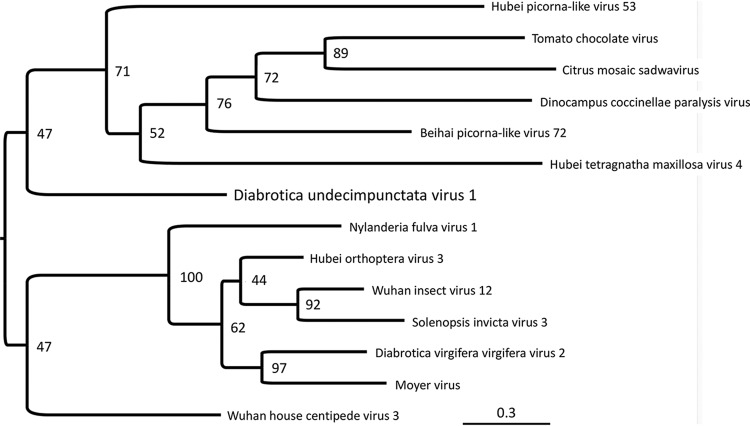
Phylogenetic analysis of DuV1 RdRP. The RdRP sequences identified by an RdRP domain search on BLAST annotation were extracted from the NCBI database. Sequences were aligned by use of MAFFT v7.4.07 with the AUTO strategy. The phylogenetic tree was constructed using the maximum likelihood (ML) method. ModelFinder implemented in IQ-TREE was used to choose the best partitioning scheme and models. ML analysis was performed using IQ-TREE with 10,000 Ultrafast bootstraps (9). Constructed trees were uploaded to Interactive Tree of Life (http://itol.embl.de) for visualization and annotation. The selected RNA viruses and their NCBI genome accession numbers are as follows: *Beihai picorna-like virus 72*, APG76793.1; *Citrus mosaic sadwavirus*, AWY04239.1; *Dinocampus coccinellae paralysis virus*, YP_009111311.1; *Diabrotica virgifera virgifera virus 2*, YP_009352233.1; *Hubei orthoptera virus 3*, YP_009336506.1; *Hubei picorna-like virus 53*, YP_009336820.1; *Hubei tetragnatha maxillosa virus 4*, YP_009336535.1; *Moyer virus*, AOC55061.1; *Nylanderia fulva virus 1*, YP_009268643.1; Solenopsis invicta *virus 3*, ACZ65579.1; *Tomato chocolate virus*, ACU01024.1; *Wuhan house centipede virus 3*, YP_009342325.1; and *Wuhan insect virus 12*, YP_009345893.1.

The genome organization of DuV1 encoding a single polyprotein with N-terminal nonstructural proteins is similar to that of *Diabrotica virgifera virgifera virus 2* (DvvV2) (3). However, the highest BLASTp score (6, 7) was an unclassified virus, *Wuhan house centipede virus 3* (GenBank accession no. YP_009342325.1) (8), with only 36% sequence coverage and 31% amino acid identity. Similar sequences were limited to nonstructural proteins. A phylogenetic tree based on the RdRP sequences of DuV1 and selected related RNA viruses highlights the novelty of this virus (Fig. 1). Based on this analysis, DuV1 represents a novel virus.

### Data availability.

The genome sequence of DuV1 is available in GenBank under accession no. MN646770. Raw reads for SRA accession no. SRX8018653 to SRX8018656 are available at BioProject no. PRJNA615920.
